# Realizing the potential of magnetic resonance image guided radiotherapy in gynaecological and rectal cancer

**DOI:** 10.1259/bjr.20180670

**Published:** 2019-05-13

**Authors:** Ingrid M White, Erica Scurr, Andreas Wetscherek, Gina Brown, Aslam Sohaib, Simeon Nill, Uwe Oelfke, David Dearnaley, Susan Lalondrelle, Shreerang Bhide

**Affiliations:** 1 Institute of Cancer Research and Royal Marsden National Health Service Foundation Trust, Sutton, Surrey, UK

## Abstract

CT-based radiotherapy workflow is limited by poor soft tissue definition in the pelvis and reliance on rigid registration methods. Current image-guided radiotherapy and adaptive radiotherapy models therefore have limited ability to improve clinical outcomes. The advent of MRI-guided radiotherapy solutions provides the opportunity to overcome these limitations with the potential to deliver online real-time MRI-based plan adaptation on a daily basis, a true “plan of the day.” This review describes the application of MRI guided radiotherapy in two pelvic tumour sites likely to benefit from this approach.

## Introduction

Multiple challenges exist in radiotherapy (RT) delivery for gynaecological and rectal targets. The target consists of volumes encompassing the primary tumour and elective nodal regions, which are difficult to visualize on CT and move independently of each other. Tumour targets are highly mobile deformable structures and are influenced by adjacent rectal and bladder filling, which is difficult to standardize throughout treatment. Substantial tumour regression can occur, which results in normal tissue falling into high dose regions, and extended field treatments are susceptible to rotational setup error.^1^ Intensity modulated radiotherapy (IMRT) reduces dose to normal tissue in gynaecological and rectal RT,^2,3^ but tight conformity and sharp dose gradients mean that adequate planning target volume (PTV) safety margins to account for geometric uncertainty are essential to avoid a geographical miss.

The current PTV margins applied to targets are based on margin recipes that aim to ensure 95% of the prescribed dose is delivered to 99% of the target volume,^4^ or 95% of the prescribed dose is delivered to 100% of the target volume in 90% of patients.^5^ Significant interpatient variability in target motion results in population-based margins that are much larger than necessary in most patients and still miss the target in a small number of cases. The alternative to large margins and increased normal tissue dose is to individualize margins and implement adaptive treatment strategies.

RT is currently planned on a single CT data set obtained at treatment simulation. This may not reflect target and organ at risk (OAR) geometry at the time of treatment delivery. Adaptive radiotherapy (ART) uses information from imaging acquired before or during treatment delivery to modify the treatment plan based on changes in individual target and OAR geometry and biology. Adaptive strategies are classified based on their timescale relative to patient treatment.^6^ Offline strategies occur between treatment fractions and typically involve a single or multiple replans. Online adaptation is based on imaging acquired immediately prior to treatment and can be used daily or intermittently. In online adaptation, tumour target and OAR interfraction changes are accounted for, which means that PTV margins can be significantly reduced.^7^ Adaptive strategies can also use information from previous treatment imaging to track the actual dose delivered to the tumour target and OARs and correct for any discrepancy between the planned and delivered dose distributions.^8^ Implementation of online adaptive strategies is limited by technical challenges, which include image quality, image registration, target and OAR segmentation, and plan reoptimization. All of which, must be performed whilst the patient remains on the treatment couch in treatment position.

Currently, image-guided RT with cone beam CT (CBCT) is limited by its ability to visualize the target and OARs and by artefact from moving gas. MRI is the gold-standard imaging modality for diagnosis and staging in gynaecological and rectal cancer and transition from CT-based to MR-based workflow in these tumour sites offers immediate advantages. MRI-guided RT (MRIgRT) will provide superior image quality at treatment planning and treatment delivery for image registration and target and OAR localization and segmentation. This will facilitate implementation of online adaptive strategies to reduce normal tissue irradiation, whilst improving target coverage. The purpose of this article is to review the advantages and challenges in the clinical application of MRIgRT in RT treatment planning and treatment adaptation using rectal and gynaecological cancers as illustrative examples.

## Search/ selection strategy

PubMed was searched using terms “Rectal Neoplasms/radiotherapy”[Mesh] or “Uterine Cervical Neoplasms/radiotherapy”[Mesh] or “Endometrial Neoplasms/radiotherapy”[Mesh] and “motion” or “adaptive” or “MR-guided” or “auto segmentation” or “auto contouring”. Search included meeting abstracts and was limited to English language. Further references were identified by cross-reference of articles. Identified studies were first screened by title and/or abstract, with further full paper screening to generate the final list of studies relevant to the scope of the present review. The last PubMed search was performed on 5 April 2018.

## Rationale for MRI-guided adaptive radiotherapy (MRIgART) in gynaecological and rectal cancer

MRI is the imaging modality of choice for diagnosis and staging in gynaecological and rectal cancer where it characterizes tumour and local macroscopic extent to inform treatment decisions, assess treatment response and detect recurrent disease.^9–11^ It is essential in identifying patients for radiation treatment, determining the radiation treatment field extent and accurate definition of the tumour target from bladder, sigmoid and small bowel.

### 1. MRI improves target localization

**Figure 1.**
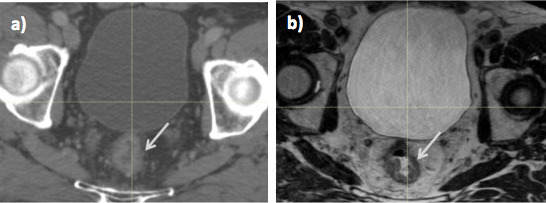
Radiotherapy planning imaging in a male patient with T3N1 rectal cancer; (a) CT and (b) MRI. On MRI, the tumour (arrow) is easily differentiated from normal rectum, which is not possible on CT.

**Figure 2.**
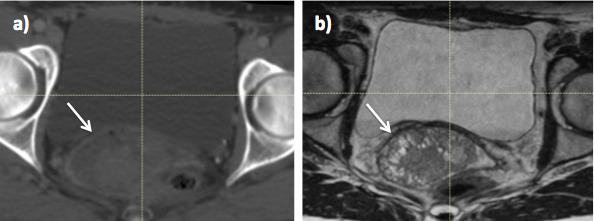
Radiotherapy planning imaging in Stage 2B cervix cancer (a) CT and (b) MRI. On MRI, the cervix tumour (arrow) is easily differentiated from normal bladder and rectum, which is not possible on CT.

Target volume delineation on the planning CT in both gynaecological and rectal tumours is difficult because it is not possible to discriminate between tumour and normal tissue. Figures 1 and 2 illustrate improved soft tissue contrast seen on MRI compared to CT for RT treatment planning in rectal and cervix cancer. Compared to CT, target volume delineation on MRI results in significantly smaller rectal and cervix volumes^12,13^ and low interobserver variability^14,15^ .Studies evaluating inter- and intraobserver variability in contour delineation on MRI in gynaecological and rectal RT are illustrated in Tables 1 and 2.^12–23^ In rectal RT, MRI delineation results in significantly reduced tumour length, width and distance of the proximal tumour edge to the anal verge *p* < 0.05.^12^ When gross tumour volume (GTV) is subdivided into tumour located in the sigmoid, rectal and anal sub regions, coverage of the CT contoured GTV was inadequate for tumours with MRI evidence of sigmoid or anal invasion.^21^

**Table 1.** Contour delineation on MRI for cervix cancer

Ref	No of patients	Structures contoured/ contour guidelines used	Method	MR sequence	Results
^13^	10	HRCTV and IRCTV GEC/ESTRO guidelines	MRI *vs* CT 1 radiation oncologist	*T*_2_W Axial	HRCTV height, thickness and total volume were similar Significant difference in width of HRCTV and IRCTV on CT compared to MRI Significant difference in volume of HRCTV treated to prescription dose or more (MRI 96%, CT 86% p ≤ 0.01)
^15^	3	GTV, nodal CTV, uterus and parametrium RTOG guidelines	Interobserver variability 12 radiation oncologists	*T*_2_W Axial	High GTV agreement (sensitivity 0.54–0.92, specificity 0.97–0.98) Moderate agreement for nodal CTV, uterus and parametrium (κ statistic 0.45–0.77 p < 0.0001) Contouring variability largest at cervix and vagina
^16^	1	GTV Cervix, uterus, vagina and parametrium RTOG guidelines	Interobserver variability 19 radiation oncologists	*T*_2_WAxial	Good sensitivity and specificity for GVT (0.84 and 0.96 respectively) Moderate agreement for cervix, uterus and vagina (κ 0.42–0.57 P < 0.001) Parametrium good specificity 0.99 but low sensitivity 0.48
^17^	19	GTV, HRCTV and IRCTV GEC/ESTRO guidelines	Interobserver variability 2 radiation oncologists	*T*_2_W Axial	No significant difference in mean volume of GTV and HRCTV *p* > 0.05 Significant difference in mean volume IRCTV *p* < 0.05 Conformity indices (range); GTV 0.6 (0.1–0.9), HRCTV 0.7 (0.4–0.8) and IRCTV 0.7 (0.5–0.8)
^18^	6	GTV HRCTV GEC/ESTRO guidelines	Interobserver variability 10 radiation oncologists	*T*_2_W Axial	Mean relative SD of 8–10% for GTV and HRCTV D90 Mean relative SD for D2cc was 5–8% for rectum and bladder, 11% for sigmoid
^19^	13	HRCTV GEC/ESTRO guidelines	Interobserver variability two experienced observers	*T*_2_W Transverse *vs* para-transverse plane	Interplane conformity index did not differ significantly between observers (0.72 *v* *s* 0.71) Interobserver conformity index between planes was not significantly different (0.79 *v* *s* 0.78) Contouring on para-transverse plane was quicker No significant difference in DVH of plans using contours from transverse or para-transverse planes
^20^	20	Elective pelvic LN volume	MRI with iron oxide particles to delineate LNs and establish pelvic LN contouring guidelines	*T*_2_W with administration of iron oxide particles	Blood vessels with a 7 mm margin, edited off muscle and bone, are a good surrogate target for the elective pelvic LN volume

GEC/ESTRO, group european de curietherapie and european society for radiotherapy and oncology; GTV, gross tumour volume; HRCTV, high risk clinical target volume; IRCTV, intermediate risk clinical target volume; LN, lymph node; RTOG, radiation therapy oncology group.

**Table 2.** Contour delineation on MRI for rectal cancer

Reference	No of patients	Structures contoured	Method	MR sequence	Results
^12^	10	GTV (entire rectal wall at level of tumour)	MRI v CT (MR <2–3/52 from CT) one radiologist	*T*_2_W sagittal	CT overestimated all tumour radiological parameters Mean MRI GTV volume 18 cm^3^ smaller than on CT *p* < 0.05 Mean MRI GTV length, max width and distance of proximal tumour to anal verge significantly less than on CT (mean reduction 3.2 cm, 0.5 cm, 2.9 cm respectively) *p* < 0.05
^21^	15	GTV	MRI V CT 1x radiologist in consultation with 1x radiation oncologist	*T*_2_ axial	Mean CT-GTV/ MRI- GTV volume ratio was 1.2cc (range 0.5–2.9) CT-GTV coverage inadequate for tumours with sigmoid or anal invasion and in the two cases this occurred there was significant underestimation of GTV on CT.
^14^	24	GTV	MRI *T*_2_ *v* *s* DWI Interobserver variation three radiation oncologists	*T*_2_W, DWI and a combination of both axial	*T*_2_ GTV volumes significantly larger than on DWI (approx. 2–3 x larger) No significant difference between observers per modality (mean conformity index 0.7 for *T*_2_W and 0.71 for DWI) Mean distance between contours *T*_2_ = 1.8 mm and DWI = 1.5 mm
^22^	27	GTV	MRI *T*_2_W *v* *s* DWI Interobserver variation two radiologists	*T*_2_W *v* *s* DWI axial	*T*_2_W MRI GTVs were slightly larger but not statistically different from DWI volumes Inter observer mean difference in volume was not improved with DWI Mean difference and 95% limits of agreement for T2W MRI and DWI GTVs were -9.8 (-55 to 35) cm^3^ and -14.8 (-54 to 24.4) cm^3^ respectively.
^23^	50	GTV	MRI pre- and post-CRT Interobserver variation two radiologists Histology reference standard for post-CRT radiology	Pre- and Post-CRT DWI and *T*_2_W MRI axial	Pre CRT MRI; Interobserver agreement for *T*_2_W and DWI was excellent (ICC 0.97) ICC all modalities; pre-CRT 0.91–0.96 and post-CRT 0.61–0.79 ROC for post-CRT volume *T*_2_W = 0.7, DWI = 0.93 and ADC = 0.54

CRT, chemoradiotherapy; DWI, diffusion weighted imaging; GTV, gross tumour volume; ICC, intraclass correlation coefficient; ROC, receiver operating characteristic.

In cervix cancer, geometric studies show that agreement between target volumes delineated on transverse and para-transverse planes of MRI is good with conformity index 0.71–0.72.^19^ In dosimetric studies, overestimation of tumour width on CT results in significant differences in the volume treated to the prescription dose or higher.^13,24^ Compared to the CT-based imaging RT workflow, MRIgRT will provide superior visualization of the target and normal tissue immediately before and during treatment delivery. Table 1 and 2 summarizes the published data for contour delineation on MRI in cervix and rectal cancer.

### 2. MRI for motion assessment

Extensive target motion occurs in gynaecological and rectal RT and has been reviewed previously.^25,26^ With RT for cervix cancer, the primary clinical target volume (CTV) includes any visible tumour, cervix, uterus, upper vagina and parametrium. The elective nodal CTV includes the pelvic and common iliac lymph nodes (LN) and the para-aortic LN in high-risk disease. Motion is largest at the uterine fundus and studies report maximum interfraction motion of over 30 mm.^27^ In one study, margins of 15 mm to the primary and nodal CTV failed in 32% of patients and margins of up to 30 mm were required to ensure coverage in 95% of fractions.^27^

With RT for rectal cancer the primary target volume includes the tumour and mesorectum, and the elective nodal volume includes the pelvic LN. The entire circumference of the rectum at the level of the tumour is included, because it is not possible to distinguish tumour from normal rectal tissue on CT. The anterior and lateral rectal wall move more than the posterior wall and motion is larger in the middle and upper rectum compared with the lower rectum.^28^ Maximum motion occurs anteriorly, particularly in the upper mesorectum, and anterior PTV margins of 24 mm in the upper mesorectum and 15 mm in the lower mesorectum have been recommended.^29,30^ Tables 3 and 4 summarize the published data for cervix and rectal interfraction target motion.^27,28,31–43^

**Table 3.** Interfraction motion in cervix cancer radiotherapy

Ref	Target measured	No of Pts	Imaging modality and Frequency	Method of measurement/ registration	Statistic used	Motion (mm)			Suggested Margins (mm)			Volume change	Bladder/ rectum correlation
						AP	LR	SI	AP	LR	SI		
^31^	Cervix	16	Weekly CT	Cervix COM Cervix contour	Mean max Range Mean max	16 5.1–25 *A* = 17 *p* = 18	8.2 4.4–14 *L* = 9.4 *R* = 7.6	21 12–33 *S* = 23 *I* = 13				Cervix volume reduced by mean 62.3% after 45 Gy	Bladder volume affects AP and SI but not lateral margins
^32^	Cervix Uterus	20	MR at baseline and weekly x5	Cervical os Uterine canal Uterine fundus Cervical os Uterine canal Uterine fund us	Grand mean Mean range	2.4 4.8 4.6 11.2 13.1 14.5		1.5 5.7 7.8 11.3 15.7 24.4	Isotropic internal margin to encompass 90% of motion was 40 mm at the fundus and 15 mm at the cervix			Significant reduction in bladder volume during RT. No systematic change in rectosigmoid volume.	Bladder volume associated with SI motion of fundus and AP motion of cervical os. Rectal volume associated with SI motion of uterine canal and cervical os.
^33^	Cervix Uterus Upper vagina	33	MR on 2 days 24 h apart	Post cervix Uterine body Upper vagina	Mean (SD) CTV–PTV margins	2.7 (2.8) 7 (9) 2.6 (3)	0.3 (0.8) 0.8 (1.3) 0.3 (1)	4.1 (4.4) 7.1 (6.8)	15 30 11	7 8	13 25 7		SI uterine motion correlated to bladder filling. AP cervix and vaginal motion related to rectal filling
^34^	GTV CTV	20	MR at baseline and weekly	GTV CTV	Margin to encompass 95% cases (internal motion)				*A* = 12 *p* = 14 *A* = 24 *p* = 17	*R* = 12 *L* = 11 *R* = 12 *L* = 16	*S* = 4 *I* = 8 *S* = 11 *I* = 8	Significant regression GTV *p* ≤ 0.001 Mean GTV 57cc week 0, 43.3cc, 32cc and 23cc at weeks 2, 3 and 4	AP shift in GTV and CTV weakly correlated with rectal vol. Significant difference in margins required if pre-treat rectum volume >70 cc.
^27^	CTV	10	Daily CBCT	COM	Mean SD Range	3 5 −9.4–18.9	−0.28 1.3 +3.3–3.5	−4.6 3.9 −15.3–3.8	Mean margin to encompass CTV motion = 15 mm, but fails in 32% Margins up to 30 mm could be required to ensure coverage in ≥95% fractions.			Mean reduction in CTV of 20% (586.4 to 469cc) Mean bladder volume relative to the planning CT −48.5 cc	Increased rectal and bladder volume associated with significant superior shifts (*p* < 0.001)
^35^	Cervix	10	Daily EPID	Cervix fiducials	Mean of mean Random error Internal motion	3.5 3.9	3.7 2.2	4.1 3.7	9.7	10.8	8.9		
^36^	Cervix	15	Portal films weekly	Radiopaque ring	Median Max	16 23	10 24	8 36				50% reduction in tumour size at 30 Gy (21 days)	
^37^	Cervix	10	Daily 2D KVI	Cervix fiducials	Mean SD Max	4.2 3.5 18	1.9 1.9 14	4.1 3.2 18					
^38^	Cervix	10	MVCT daily	Cervix contour Uterus contour 95% margin for internal motion and setup	Mean SD Mean SD	*A* = 0.4 P=-3 *A* = 10.1 *p* = 6.9 *A* = 3.3 *p* = 0.3 *A* = 11.9 *p* = 11.7	*L* = −3.5 *R* = 0.2 *L* = 4.9 *R* = 4.5 *L* = 0.7 *R* = −0.6 *L* = 8.1 *R* = 7.5	*S* = 2.2 *I* = 0.5 *S* = 8 *I* = 5 *S* = 6.1 *I* = 5 *S* = 11.6 *I* = 11.2	*A* = 17 *p* = 12 *A* = 19 *p* = 19	*R* = 8 *L* = 9 *R* = 13 *L* = 13	*S* = 15 *I* = 9 *S* = 20 *I* = 19	Significant reduction in mean cervix volume (106 cc pre-treatment to 74 cc last week of treatment)	Average bladder volume reduced from 156 cc in wk1 to 88 cc in the last week (*p* < 0.01).
^39^	Cervix	20	MRI baseline and weekly x5	GTV Cervix Uterus Upper vagina	Euclidean vector displacement	1.2 ± 0.4 (0.5–3) 1.1 ± 0.3 (0.5–2.8) 1.7 ± 0.2 (0.5–4.5) 0.7 ± 0.3 (0.3–1.3)			15 mm GTV to PTV margin covered the GTV to >98% of prescription dose			The relative reduction in the GTV from baseline to the end treatment was 48–96%.	Individually, the planned dose was not the same as the simulated delivered dose

COM, centre of mass; CTV, clinical target volume; 2D, two-dimensional; EPID, electronic portal imaging device; GTV, gross tumour volume; KVI, kilovoltage imaging; MVCT, megavoltage CT; PTV, planning target volume; RTP, radiotherapy planning; SD, standard deviation.

**Table 4.** Inter- and Intrafraction motion in rectal cancer

Ref	Target measured	No of Pts	Imaging modality and frequency	Method of measurement/ registration	Statistic used	Motion (mm)			Suggested margins (mm)			Volume change	Other
						AP	LR	SI	AP	LR	SI		
^40^	GTV Rectum Mesorectum	17	RTP CT Wk1, 3 and 5	Displacement of points on GTV, rectum and mesorectum surface	Mean (SD) GTV Rectum Mesorectum	0.7 (3.1) 1.1 (5.1) 1.1 (2.7)	1.2 (2.8) 0.2 (4.5) 0.3 (2.2)	4.2 (3.6)	*A* = 14 *p* = 7 *A* = 8 *p* = 9 *A* = 7 *p* = 6	*L* = 7 *R* = 8 *L* = 8 *R* = 8 *L* = 5 *R* = 4	*S* = 16 *I* = 12		Greatest motion of rectum in upper 1/3 No correlation of motion direction and bladder filling
^41^	Mesorectum	10	Helical MVCT before and after treatment x2/week	Contour displacement by bony landmarks	Mean (SD) Margins for intrafraction motion and setup	A=-2(6.8) *p* = −0.4 (3.8)	*L* = −1.6 (4.2) *R* = 0.1 (4)	*S* = −3.2 (5.6) *I* = −3.2 (6.8)	*A* = 11 *p* = 7	8	*S* = 10 *I* = 12		If new margins applied instead of standard 1 cm margins, there would be an average decrease in PTV by 21.5% (SD, 1.45%).
^28^	Rectum	16	CBCT D1-3, then weekly GTV to PTV margin	Upper rectum Mid rectum Low rectum	Mean of mean Mean of SD Mean of mean Mean of SD Mean of mean Mean of SD	A= −4 *p* = −0.1 *A* = 7.4 *p* = 4.2 A= −1 *p* = −0.1 *A* = 7 *p* = 3.6 *A* = 1.8 *p* = 1.2 *A* = 4.2 *p* = 4.7	*L* = 1.3 *R* = −2.8 *L* = 6.9 *R* = 5.2 *L* = −0.4 *R* = 0 *L* = 5.1 *R* = 4.1 *L* = 0.1 *R* = 0.0 *L* = 3 *R* = 3		*A* = 17 *p* = 14.4 *A* = 16.7 *p* = 14.9 *A* = 14.2 *p* = 16	*L* = 4.2 *R* = 4.2 *L* = 11 *R* = 10.3 *L* = 9 *R* = 10.1		No significant change in rectal volume on CBCT compared to baseline CT	No relationship between rectal and bladder volume and time Significant day to day bladder volume variation
^42^	CTV Rectum	10	Weekly RTP CT CTV Rectum	At AV 5.5 cm from anus 9 cm from anus At anus 4.5 cm from anus 9 cm from anus	CTV SD of motion Rectum SD of motion	*A* = 3–4 *A* = 6 *A* = 10 *p* = No motion *p* = 4 *p* = 7 *p* = 2 A= ‘very similar to CTV’	No motion observed Motion similar to CTV, *i.e*. no motion						Motion dependent on location in pelvis Increased motion of CTV at ≥5.5 cm from anus caused by bladder filling Biggest motion at 10 cm from anus The biggest difference in CTV volume between a full and empty bladder was 51 cm^3^
^43^	Mesorectum	63	Repeat RTP CT LCRT daily CT for first week and then weekly. SCRT cohort daily CT	LCRT Upper Mesorectum Lower mesorectum SCRT Upper Mesorectum Lower Mesorectum	PTV margins for 95% prescribed dose to 90% patients				*A* = 24 *p* = 7 *A* = 15 *p* = 7 *A* = 32 *p* = 7 *A* = 18 *p* = 7	*L* = 7 *R* = 7 *L* = 7 *R* = 7 *L* = 7 *R* = 7 *L* = 10 *R* = 10	*S* = 10 *I* = 10 *S* = 10 *I* = 10 *S* = 10 *I* = 10 *S* = 10 *I* = 10	Significant reduction in rectal volume in LCRT by 35% Reduced bladder volume during RT	Significant reduction in rectal volume resulted in 5 mm post shift of upper ant CTV

AV, anal verge; CTV, clinical target volume; GTV, gross tumour volume; LCRT, long-course chemoradiotherapy; MVCT, megavoltage CT; PTV, planning target volume; RTP, radiotherapy planning; SCRT, stereotactic conformal radiotherapy; SD, standard deviation.

**Figure 3.**
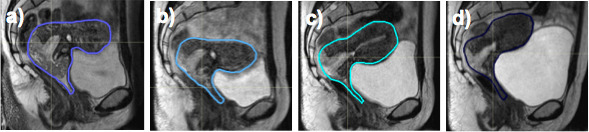
Changes in clinical target volume position during cervix radiotherapy as seen on MRI at (a) week 0, (b) week 2, (c) week 3 and (d) week 4.

Bladder and rectal filling influence target motion in gynaecological and rectal RT. With cervix treatment, bladder volume is correlated with superior/inferior uterine motion and rectal volume is correlated with cervix and vaginal anterior/posterior motion.^33^ With rectal RT, deformation of the mesorectum is largely driven by changes in rectal volume.^29^ In both cervix and rectal RT, there is significant interpatient variation in bladder volume despite bladder filling protocols, and both bladder and rectal volumes reduce during treatment.^27,28,34,44^ Laxatives may not significantly reduce target anterior/posterior motion from rectal volume variation, because passage of gas can still cause significant target displacement.^37^ Figure 3 illustrates CTV positional changes related to bladder volume as seen on CBCT during cervix RT. MRIgART will facilitate implementation of margin reduction through adaptive strategies that account for these geometric changes.

### 3. MRI for anatomical response assessment and dose escalation

Significant tumour regression is observed during cervix and rectal RT.^31,34,45,46^ In 20 cervix patients having weekly MRI during chemoradiotherapy (CRT), average tumour volume reductions of 59.6% at week 4 were observed, which resulted in increased uterine motion, substantial changes in tumour position and movement of normal tissue, particularly small bowel, into the high dose region.^47^ Repeat MRI and planning after delivery of 30 Gy found that a second IMRT plan significantly reduced the volume of bowel irradiated if the primary GTV decreased >30 cc.^47^

In a study of 15 rectal cancer patients, mean tumour regression of 46.3% was seen on MRI by week 5 of CRT and regression was fastest in the first 3 weeks of treatment.^45^ A further study in 13 patients found that the majority of patients who had a good response to treatment had volume reduction and fibrotic changes during weeks 1–3.^46^ There is a move towards organ preservation in rectal patients with a complete radiological response to spare morbidity from surgery.^48^ Patients who respond to CRT are more likely to benefit from dose escalation to increase the rate of pathological complete response (pCR)^46^ and early assessment to identify these patients is therefore important. Response to neo-adjuvant CRT is dose-dependent with dose escalation of >60 Gy resulting in increased rates of pCR and acceptable toxicity.^49^ Tumour boost volume delineation on the initial RT planning CT does not take account of tumour regression during treatment. Repeat imaging during treatment could help select patients who would benefit from radiation dose escalation and would produce more accurate and smaller boost volumes, facilitating increased tumour dose without increased OAR dose and toxicity.^50^

**Figure 4.**
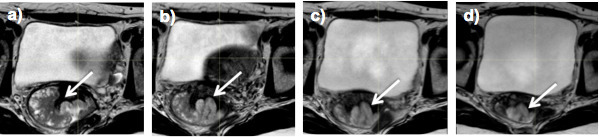
Changes in cervix tumour volume (arrow), as seen on weekly MRI during treatment at (a) week 0, (b) week 2, (c) week 3 and (d) week 4.

**Figure 5.**
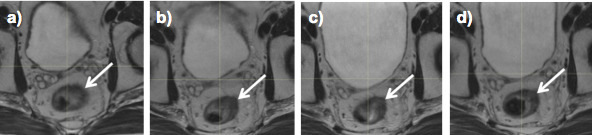
Changes in rectal tumour volume (arrow), as seen on weekly MRI during treatment at (a) week 0, (b) week 2, (c) week 3 and (d) week 4.

Figures 4 and 5 illustrate changes in cervix and rectal tumour volume as seen on weekly MRI during RT.

### 4. MRI for biological response prediction and dose delivery assessment

Functional MRI with diffusion-weighted imaging (DWI) and dynamic contrast enhancement (DCE) may predict biological response in rectal and cervix RT and identify patients for dose escalation.^14,51^

MRI has potential to act as a biomarker, identifying good and poorly responding tumours to select patients for dose adaptation in order to improve treatment outcomes.^52–55^ Studies suggest that DWI can predict pathological complete response early in rectal RT,^53,54,56,57^ but there are limitations to the current evidence preventing its routine implementation in patient selection for dose escalation. Most studies were small and did not prospectively determine MRI criteria to differentiate between complete and non-complete response to treatment. Retrospective identification of these parameters introduces selection bias. There was variability in the time points at which imaging was acquired and surgery was performed. For example, patients classified as achieving a non-pCR at 6 weeks following CRT may have been classified as a pCR if surgery was performed at a later date and meta-analysis reports 6% increase rate of pCR with an interval of greater than 6 weeks from the end of preoperative CRT.^58^

In cervix RT, DCE and DWI MRI may predict response to CRT and identify patients for dose escalation.^51^ Increasing apparent diffusion coefficient (ADC) values from DWI acquired during treatment can detect early signs of treatment response.^55^ DCE MRI during treatment detects tumour perfusion.^59^ Persistently low perfusion during CRT is correlated with treatment failure and patients with increases in perfusion during CRT have better outcomes.^59^ This could identify patients for dose escalation to hypoxic regions, which should increase tumour shrinkage prior to brachytherapy, which we know improves local control.^60^ There was however, no technical standardization in these studies, which limits assessment of reproducibility and generalizability. The optimal time to assess biological response and adapt treatment based on these finding has yet to be determined.

MRIgRT will also provide quantitative knowledge of the actual delivered dose and the impact of radiation dose on tumour and normal tissue. This would enable dose compensation strategies and tumour and normal tissue radiobiological modelling.

## Adaptive radiotherapy (ART) strategies

### 1. Target volume modification based on individual internal motion

PTV modification based on data from setup and internal target motion acquired from planning or previous treatment, allows safe reduction of generic population based margins. This is also referred to as a composite volume technique. The range of target motion is modelled during the planning stage or first treatments to generate an internal target volume (ITV). The treatment plan is optimized offline and applied to subsequent treatments. Individualized ITVs in cervix RT account for the range of cervix and uterine motion with variable bladder volume and may be based on variable bladder filling CT scans acquired at simulation, or using bladder geometry as a predictive tool.^61,62^ Compared to population-based margins, individualized margins reduce CTV–PTV margins by 48% (±6%), and bladder and rectal volume within the PTV is reduced by 5–45% and 26–74% respectively.^62^

For rectal cancer, an average CTV can be acquired from the radiotherapy planning (RTP) CT and repeat CTs during the first week of treatment.^30^ Adaptation after day 4 resulted in a 7 mm reduction in the maximum required PTV margin from 24 to 17 mm and a significant reduction in PTV and dose to the small bowel.^30^

### 2. Online plan selection strategy

Online plan selection uses imaging acquired at treatment to select a plan from a library of treatment plans generated from multiple PTVs. In cervix RT, evaluated strategies include a plan library using individualized PTVs based on CTV position at different bladder volumes, or PTVs created by the application of incremental margins to the CTV as seen on RTP CT acquired with a full bladder.^62,63^ Compared to a standard population margin approach, plan selection results in significantly better target coverage and OAR sparing.^62–64^ Adaptation based on variable bladder filling CTVs enables reductions in PTV margins from 38 to 7 mm and better CTV D98% > 95% in comparison to the non-ART approach where 17% of treatment fractions have inadequate target coverage.^62,64^ When using an incremental margin approach, a 5 mm margin of the day plan could be used in 25% of fractions.^63^ Libraries based on variable bladder filling do not account for rectal filling variation or the passage of gas, which are difficult to predict and can significantly influence cervix motion.^65^

In rectal cancer, target motion is influenced more by rectal than bladder filling, so a library of plans strategy based on variable bladder volumes is not appropriate. Instead plan selection has been based on plans with variable PTV margins between −25 and +25 mm applied to the anterior CTV, which is where largest variation is seen.^66^ This reduced dose to the bladder and small bowel OARs, although the absolute reductions were small.^67^ Plan selection in rectal RT is feasible with good plan selection consistency between observers of 75%.^66^ Plan selection in both cervix and rectal radiotherapy is being implemented clinically, but is limited by the image quality of CBCT. MRIgRT would facilitate target and OAR localization for online plan selection.

### 3. Plan reoptimization

The optimal strategy to account for target and OAR motion and deformation, anatomical and biological response, is to generate a new plan with full reoptimization. This determines the dose distribution based on target and OAR geometry and/or physiology at the time of treatment delivery.^6^

A number of planning studies in cervix RT have simulated the benefit of online replanning.^7,68,69^ One study of 33 patients compared a 3 mm PTV margin plan without replanning, with an automated weekly replan on real-time patient geometry as seen on MRI.^68,69^ Pre-treatment optimization criteria were automatically reapplied to replans without any physics planner intervention. Without replanning, there was a significant reduction in accumulated dose to the primary CTV, with nine patients failing D98% > 95%.^68^ In patients who were replanned, there was a reduction in CTV between 8 and 68% (median 39%) and the D98 CTV constraint was met in all patients.^68^ There was no difference in dose to OARs, which might move with the target and remain in the high dose region. This may lead to increased OAR dose in patients where OAR movement is related to the target compared to patients where the OARs move independently.^68,69^

A study in 14 cervix patients used 15 mm PTV margins and replanning based on target and OAR geometry on MRI after 30 Gy.^47^ There was a reduction in OAR dose with replanning, but in this study the replans were interactively optimized to reflect new anatomy.^47^ A planning study to simulate the benefit of online MRIgRT replanned using weekly MRI in 11 patients receiving IMRT for cervix cancer with 4 mm PTV margins.^7^ This was compared to plans based on the pre-treatment MRI with primary and nodal PTV margins of 15 and 10 mm.^7^ There was a significant reduction in the dose to the bladder, rectum, sigmoid, and small bowel with online replanning.^7^

### 4. Dose compensation

Adaptation using dose tracking allows reduction in PTV margins because variations in the dose delivered to the CTV compared to the planned dose, can be compensated for in subsequent fractions. The pre-treatment imaging, together with any setup correction applied, is used to determine target and OAR position and the dose delivered at each treatment fraction. This is non-rigidly registered to the planning CT to model anatomical motion and deformation and allows calculation of the accumulated delivered dose. The treatment plan can then be reoptimized to compensate for any problems with dose coverage or to account for adaptation of treatment goals.

Lim et al looked at pre-treatment and weekly MRI in 30 cervix IMRT patients using a 3 mm PTV margin and dose accumulation.^70^ They modelled an anatomical driven approach with a single offline replan mid-treatment to account for tumour regression, and a dosimetrically triggered approach if the estimated accumulated D98 to the GTV or primary CTV was low. Without replanning, there was insufficient target coverage in 27% of patients. The anatomical approach improved target coverage and reduced OAR dose, but there were still three patients with insufficient target coverage. Dosimetrically triggered replanning resulted in target coverage in all patients, but no difference in the accumulated OAR dose.^70^ Deformable registration is not consistently accurate and validation is difficult. In deformable registration for dose accumulation, particular caution must be taken when tumours have undergone mass change and in areas with sharp dose gradients.

## INTEGRATION OF MRI INTO RADIOTHERAPY AND ITS CHALLENGES﻿

MRI can be integrated into RT workflow in a variety of ways. In a CT-MRI simulation workflow, the MRI is used for contour delineation at radiotherapy treatment planning (RTP) and the CT provides a robust geometric representation of the patient, an electron density map required for dose calculation and a reference image for patient set up during standard treatment. Any error in image registration will however lead to a systematic geometric error throughout patient treatment.^71^ MRI-only simulation reduces potential for image registration error at RTP, but the challenges of geometric distortion and lack of electron density information and material properties inherent to MRI need to be addressed. MRI for RT treatment localization, planning and verification have different demands to those acquired for diagnosis and staging. Specific solutions are required. The main differences relate to patient positioning, image acquisition and sequence parameters and the need for geometric accuracy (Table 5).

**Table 5.** Different demands of MRI acquired for diagnostic and radiotherapy purposes in cervix and rectal cancer

	**MRI for diagnosis**	**MRI for radiotherapy**
**Couch**	Soft, often concave Maximized for patient comfort	Needs to be flat, the same as in RT delivery
**Patient positioning**	Comfortable Supine	As for RT delivery Supine
**Immobilization devices**	None	Combifix knee support to stabilize pelvis
**Bowel artefact management**	IM Buscopan Anterior abdominal wall compression Saturation bands	IM Buscopan may be used in MRI simulation but may not be acceptable during daily treatment within MRI treatment workflow
**Bladder status**	Empty	Full
**Coil placement**	Pelvic coil centred on tumour	Anterior coil supports prevent distortion of external body contour Customized MR simulators may incorporate posterior coils into a flat couch
**Field strength**	Increasing strength improves signal to noise, but is more expensive and requires more room	Increasing field strength increases geometric distortion
**Coverage**	High resolution FOV limited to tumour	High resolution FOV must encompass entire tumour target Sequences including external body contour required for dose calculation
**Preferred** **sequence**	2d *T*_2_W high resolution at tumour with ≤3 mm slice thickness, and voxel size <1 mm Imaging plane perpendicular to the rectum or cervical canal	*T*_2_W 3*d* < 1 mm isotropic voxel size for target delineation Imaging plane true axial acquired perpendicular to the system
**Geometric accuracy**	Less important	Essential to localize the target
**Electron density/ material composition information**	Not required	Not required in a CT/ MRI combined workflow, but essential in MR-only simulation and MR treatment workflow

FOV, field of view;RT, radiotherapy.

A number of MRIgRT technologies are in active development, integrating MRI with external beam RT delivery, providing MRI data immediately before and after treatment, and simultaneously with treatment delivery.^72–75^ They differ in their imaging and treatment adaptation capabilities and their approach to tackling the technical challenges of magnetic and radiofrequency interference and treatment beam transmission through the magnet. Table 6 summarizes the different systems, each presenting advantages and disadvantages.^72–77^ The MRIdian system (ViewRay Inc, Oakwood Village, OH) has treated over 300 patients since 2014 and integrates a 0.35 Tesla (T) magnet with either three multileaf collimator (MLC)-equipped Cobolt-60 heads, or a 6 MV linac.^73,78^ The Elekta Unity MR-linac solution (Elekta AB, Stockholm, Sweden) started treating patients in 2017 under pre-CE mark clinical trial protocol. It integrates a 7 MV linac with a high field 1.5 T MRI system from Philips, which uses technology similar to the Philips Ingenia diagnostic systems.^72^ Lower magnetic field solutions benefit from a reduction in image artefacts and patient related geometric distortion, and lower energy deposition by the radiofrequency pulses. Higher field solutions benefit from enhanced signal to noise, which improves spatial and temporal resolution and functional imaging capabilities.

**Table 6.** MRI guidance radiotherapy systems

	**Elekta Unity MR Linac** ^72,76^	**ViewRay MRIdian Cobalt 60** ^73,77^	**ViewRay MRIdian Linac**	**Australian MRI-Linac** ^74^	**Canadian Aurora Magnet X MR Linac** ^75^
**Magnet**	1.5 T closed	0.35 T split bore	0.35 T split bore	1.0 T split bore	0.5 T biplanar rotating geometry
**Radiotherapy source**	7 MV	3 Cobalt-60 heads	6 MV	6 MV	6 MV
**MLC effective leaf width at isocentre**	0.72 cm	1.05 cm	0.83 cm		
**MLC maximum leaf speed**	6 cm/sec	2.0 ± 0.1 cm/sec	>2 cm/ sec		
**Magnetic field orientation to delivery**	Perpendicular	Perpendicular	Perpendicular	Perpendicular and parallel	Perpendicular and parallel
**Bore Size**	70 cm	70 cm	70 cm	50 cm	60 cm
**Magnetic field homogeneity**	≤2.0 ppm over 50 × 50×45 cm^3^	<25 ppm over 45 cm DSV	<25 ppm over 45 cm DSV		
**Maximum imaging field of view**	50 cm DSV	50 cm DSV	50 cm DSV		
**Maximum treatment field size**	57.4 × 22 cm^2^	27.3 × 27.3 cm^2^	27.4 × 24.1 cm^2^		
**4D capabilities**	Yes	Yes	Yes	No	No
**Functional imaging**	Yes	Yes	Yes	No	No
**Treating patients**	Yes	Yes	Yes	No	No
**CE Marked/ FDA approved**	Yes	Yes	Yes	No	No

DSV, diameter of spherical volume; MLC, multileaf collimator; Ppm, parts per million.

## TECHNICAL CHALLENGES IN THE REALIZATION OF REAL-TIME MRIGART

Generation of a new treatment plan based on target and OAR geometry or biology at the time of treatment delivery is the ultimate goal of MRIgART. The main challenge is achieving this in a short amount of time with the patient on the treatment couch. Its clinical implementation is limited by;

1. Requirement for robust automated real-time registration of the newly acquired MRI with the images used for treatment planning.
2. Requirement for electron density data necessary for dose calculation.
3. Target and OAR segmentation on the new MRI.
4. Plan reoptimization and dose calculation.
5. Quality assurance of the newly generated plan.

In the first clinical applications of MRIgART using the Elekta Unity MR-Linac (Elekta AB, Stockholm, Sweden) and the MRIdian system (ViewRay, Oakwood Village, OH), MRI are acquired immediately before treatment and registered to the reference planning MRI and planning CT using deformable registration.^79,80^ Electron density information from the reference planning CT is then transferred to the MRI of the day using the deformation map.^79,80^

The standard treatment-planning process requires segmented contours and generates the desired dose distribution from scratch. This is achieved through iterative optimization, driven by defined objective functions set by the planner, which specify the dose–volume constraints for tumour targets and OARs. The planner then fine-tunes the objective functions and repeats the optimization process to further improve the treatment plan by trial and error. This takes too long to be feasibly implemented in real-time MRIgART and faster automated replanning strategies are required.

Segmentation of target and OARs on the daily image is a major challenge in online replanning. Manual segmentation is time consuming and susceptible to inter- and intraobserver variability. Mean time required to manually delineate the pelvic nodal CTV alone is over 30 min, and automated strategies are necessary to reduce segmentation time and improve structure definition.^81^ Autosegmentation without prior knowledge uses imaging properties such as voxel intensities and gradients.^82^ Alternative strategies incorporate prior knowledge into the segmentation process to improve accuracy and reproducibility and include atlas-based segmentation, statistical shape models, machine learning and hybrid strategies.^82^

In atlas-based autosegmentation, an atlas of manually contoured structures is used to propagate structures onto a new data set using deformable registration voxels transformations.^83–85^ Use of multiple atlases further improves accuracy.^86^ Cervix target segmentation on MRI using machine learning results in mean sensitivity and specificity of 85–93%^87^ and is faster than atlas based strategies.^88^ Accuracy of autosegmentation is not perfect and visual verification is still required. In MRIgART using both the Elekta Unity MR-Linac and the ViewRay MRIdian systems, target and OAR contours are transferred to the online MRI from the reference image using deformable registration and are then checked and manually edited if necessary by a clinician.^78,80,89^

Daily plan reoptimization does not need to start from scratch and many components of the new plan can be extrapolated from the original fully optimized plan. Plan modification with aperture morphing reduces the number of steps in reoptimization.^90^ Segment aperture morphing adjusts the beam segment shape of the MLC, based on the new target position and shape, as seen in the projection from the beam’s eye view of each treatment beam. Segment weight optimization can then be applied to improve dosimetry.^90^ More complex aperture morphing methods rely on deformable registration.^91,92^

Plan adaptation based on previous knowledge from the original plan can also speed up the process. Gradient maintenance strategies maintain the same dose gradient around the target, towards the OARs, as in the original treatment plan.^93^ This requires segmentation of the new target but not segmentation of OARS. It may not be suitable for the larger target volumes seen in gynaecological and rectal RT. Interactive dose shaping is based on contoured structures and enables direct manipulation of the initial plan isodose surface shape or the dose to individual voxels.^94,95^ Advances in computer power, both graphical processing units and modified central core processing units, can now reduce the time of plan optimization and dose calculation from minutes to seconds.^96,97^ Commercial treatment planning systems incorporating advances in adaptive planning are now becoming available.

Plan approval and quality assurance (QA) in real-time MRIgART is challenging. Automation of image acquisition and registration, target and OAR segmentation, treatment dose calculation and adaptive planning optimization is essential in implementing online MRIgART, but creates additional problems. The detailed plan reviews and QA process that occur at pre-treatment during standard RT are not appropriate. Limiting physician plan approval to when plan quality is less than the original treatment plan would improve efficiency. Conventional patient specific QA approaches insert physical phantoms in the treatment beam, which cannot be used with the patient on the treatment couch. An alternative solution is to send the treatment plan to an independent dose calculation engine to verify that the dose distributions agree.^98^

Delivery of MRIgRT with the ViewRay MRIdian Cobalt 60 was feasible in 11 rectal patients receiving neoadjuvant chemoradiation with IMRT and simultaneous integrated boost.^99^ Daily MRI were acquired for patient setup and verification, and all patients completed treatment. The ViewRay MRIdian has also been used for imaging and RT planning in brachytherapy for cervical cancer.^100^ No studies have yet been published for MRIgRT delivery in cervix external beam RT.

## Conclusions

MRIgRT in rectal and gynaecological RT will improve all aspects of the treatment workflow. Its most exciting application in gynaecological and rectal RT will be to refine GTV to CTV definition, increased accuracy and precision of target localization for treatment verification and implementation of adaptive strategies to personalize the therapeutic approach. This will facilitate reduced PTV margins and normal tissue irradiation whilst maintaining target coverage. Together with dose adaptation, this will translate into improved tumour control and reduced toxicity for patients. Optimal adaptive strategies need to be determined and challenges remain for the implementation of MRIgART clinical workflow. But technology is exponentially increasing and the ability to personalize and intensify treatment with MRIgART at these tumour sites is no longer an improbable blue-sky ideology but is now within reach.
